# Spatiotemporal specificity of contrast adaptation in mouse primary visual cortex

**DOI:** 10.3389/fncir.2013.00154

**Published:** 2013-10-03

**Authors:** Emily E. LeDue, Jillian L. King, Kurt R. Stover, Nathan A. Crowder

**Affiliations:** Department of Psychology and Neuroscience, Dalhousie UniversityHalifax, NS, Canada

**Keywords:** adaptation, mouse vision, primary visual cortex, sinusoidal gratings, pattern-specificity, electrophysiology, context

## Abstract

Prolonged viewing of high contrast gratings alters perceived stimulus contrast, and produces characteristic changes in the contrast response functions of neurons in the primary visual cortex (V1). This is referred to as contrast adaptation. Although contrast adaptation has been well-studied, its underlying neural mechanisms are not well-understood. Therefore, we investigated contrast adaptation in mouse V1 with the goal of establishing a quantitative description of this phenomenon in a genetically manipulable animal model. One interesting aspect of contrast adaptation that has been observed both perceptually and in single unit studies is its specificity for the spatial and temporal characteristics of the stimulus. Therefore, in the present work we determined if the magnitude of contrast adaptation in mouse V1 neurons was dependent on the spatial frequency and temporal frequency of the adapting grating. We used protocols that were readily comparable with previous studies in cats and primates, and also a novel contrast ramp stimulus that characterized the spatial and temporal specificity of contrast adaptation simultaneously. Similar to previous work in higher mammals, we found that contrast adaptation was strongest when the spatial frequency and temporal frequency of the adapting grating matched the test stimulus. This suggests similar mechanisms underlying contrast adaptation across animal models and indicates that the rapidly advancing genetic tools available in mice could be used to provide insights into this phenomenon.

## Introduction

Our perception of the world around us, and the neural activity underlying this experience, is strongly dependent on the recent stimulus history. In the visual system, there is evidence for a number of self-calibration mechanisms that rapidly adapt visual processing according to the prevailing attributes of the stimulus being viewed (Carandini, 2000). Contrast adaptation has been used extensively to study this form of short-term plasticity. In psychophysical studies, prolonged viewing of a high-contrast pattern can produce a perceived fading of the adapting stimulus and reduce sensitivity to low contrasts, but it can also improve sensitivity and discrimination around the adapting contrast (Blakemore and Campbell, 1969; Greenlee and Heitger, 1988; Foley and Chen, 1997; Abbonizio et al., 2002). Primary visual cortex (V1) neurons have sigmoidal contrast response functions when spike rate is plotted as a function of stimulus contrast, and contrast adaptation has been shown to shift the most sensitive part of the curve toward the adapting contrast (Movshon and Lennie, 1979; Ohzawa et al., 1982, 1985; Sclar et al., 1989; Bonds, 1991; Ibbotson, 2005). A case has also been made that contrast adaptation (and similar processes) must be incorporated into models of V1 to better predict the responses of real neurons to natural stimuli (Carandini et al., 2005). Thus, there is converging evidence that contrast adaptation is a fundamental process that the visual system uses to make moment-to-moment adjustments in its sensitivity to incoming input.

Both psychophysical observations and single unit recording studies in V1 indicate that contrast adaption is pattern-specific such that its magnitude can depend on the spatial frequency (SF), temporal frequency (TF), or orientation of the adapting and test stimuli (Blakemore et al., 1973; Vautin and Berkley, 1977; Movshon and Lennie, 1979; Albrecht et al., 1984; Ohzawa et al., 1985; Saul and Cynader, 1989a,b; Snowden and Hammett, 1996; Müller et al., 1999). This pattern-specificity has been used to constrain possible mechanisms underlying contrast adaptation. For example, both psychophysical and V1 data indicate that contrast adaptation is strongest when the SF of the adapting stimulus matches the test stimulus (psychophysics: Blakemore and Campbell, 1969; Blakemore and Nachmias, 1971; Blakemore et al., 1973; Snowden and Hammett, 1996; neurophysiology: Movshon and Lennie, 1979; Ohzawa et al., 1985; Saul and Cynader, 1989a), but this SF specificity must develop in the cortex because contrast adaptation in the lateral geniculate nucleus (LGN) does not appear to be SF specific (Duong and Freeman, 2007).

Several cellular and circuit mechanisms have been proposed to play a role in contrast adaptation (for a review see Kohn, 2007), but understanding of the cause of contrast adaptation remains incomplete. Several useful genetic tools available in mice could provide another avenue to explore contrast adaptation, but baseline conditions must first be established in this species to make any genetic manipulation related to contrast coding interpretable. Several recent studies of mouse V1 have revealed similarities between mice and higher mammals, including tuning for spatial and temporal frequencies, selectivity for orientation and direction, and the presence of simple and complex cells (Niell and Stryker, 2008; Gao et al., 2010; Van den Bergh et al., 2010). However, contrast adaptation in mouse V1 has been reported in only two studies (Niell and Stryker, 2008; Stroud et al., 2012). Stroud et al. (2012) investigated the orientation specificity of contrast adaptation, but the other two aspects of the pattern-specificity of contrast adaptation that have been so important for linking electrophysiological studies in higher mammals to human psychophysical observations, namely specificity for SF and TF, remain unexplored. Therefore, we examined the spatiotemporal specificity of contrast adaptation in mouse V1 using a top-up adaptation protocol that was comparable with previous studies in cat and monkey (Movshon and Lennie, 1979; Duong and Freeman, 2007; Dhruv et al., 2011). We also used dynamic contrast ramp stimuli of varying SF and TF to obtain rapid measures of contrast adaptation with a wide variety of adaptors (Crowder et al., 2008; Stroud et al., 2012).

Mouse V1 neurons showed robust contrast adaptation when the adapting grating matched the neuron's preferred stimulus, which confirms earlier findings (Stroud et al., 2012). Furthermore, in the top-up protocol contrast adaptation was diminished or absent when the SF or TF of the adaptor did not match the neuron's preference, indicating that mouse V1 neurons show adaptation specificity similar to that observed in cats and primates. Adaptation observed in the contrast ramp experiments was also pattern-selective, but maximal adaptation often occurred at slightly higher-than-preferred SFs, indicating that the exact properties of the contrast adaptation observed depends on the nature of the testing protocol.

## Materials and methods

### Anesthesia and surgical procedures

The experimental procedures reported herein conform to the guidelines established by the Canadian Council on Animal Care, which were approved by the University Committee on Laboratory Animals at Dalhousie University. Electrophysiological recordings were made from 25 adult male C57 BL/6 J mice weighing between 20 and 30 g, which were purchased from Jackson Laboratories (Bar Harbor, Maine). In early experiments, mice (*n* = 15) were sedated with chlorprothixene (5 mg/kg ip; Sigma, St. Louis, MO), and then anesthetized with urethane (0.5–1.2 g/kg ip; Sigma). If needed, a small dose of ketamine (20 mg/kg ip; Wyeth) was given to accelerate descent to the surgical plane of anesthesia, and allow a tracheotomy to be performed quickly (see Moldestad et al., 2009 for details). Mice were left free-breathing throughout the experiment and a tube located in front of the mouse delivered oxygen (0.1 L/min) to supplement room air. In later experiments, mice (*n* = 10) were sedated with chlorprothixene (5 mg/kg ip) and anesthetized with isoflurane delivered through a customized nose cone (2.5% during induction, 1.5% during surgery, and 0.4–1% during recording), which decreased preparation time by eliminating the need for a tracheotomy. Gas anesthesia did not appear to affect the frequency of encountering responsive units, and produced no significant differences in the tuning strength or selectivity of recorded units (assessed with discrimination indices, see *Initial data analysis* below; two-sample *t*-tests, *p* > 0.2 for all; c.f. Kaneko et al., 2012). For all mice, body temperature was maintained at 37.5°C with a heating pad, and their corneas were protected by frequent application of a thin layer of optically neutral silicone oil (30000 cSt; Sigma). The skull was stabilized in a stereotax, and a craniotomy (~1 mm^2^) was made over the monocular retinotopic representation in primary visual cortex (~0.8 mm anterior and 2.3 mm lateral to lambda; Paxinos and Franklin, 2001). Recordings were made using either glass micropipettes (2–5 μm tip diameter, filled with 2 M NaCl) or carbon-fiber in glass microelectrodes (0.6–1.5 MΩ impedance). Electrode depth was controlled using a micromanipulator (FHC, Bowdoin, ME). Extracellular signals from individual units were amplified (Xcell 3+, FHC) and filtered (bandpass: 50–2000 Hz) before being digitized (Cambridge Electronic Design Power1401 with Spike2, Cambridge, England). Acquired signals were sampled at 40 kHz, and online analysis was performed on triggered TTL pulses with Spike2, but subsequent analysis was done offline.

### Visual stimuli

Upon isolation of a visually responsive unit, the receptive field (RF) was mapped using hand-driven light bars and spots. Quantitative testing was then performed with custom computer generated visual stimuli programmed in MatLab (MathWorks, Natick, MA) using the Psychophysics Toolbox extension (Brainard, 1997; Pelli, 1997), and presented on a calibrated CRT monitor (LG Flatron 915FT plus 19” display, 100 Hz refresh, 1024 × 768 pixels, mean luminance = 30 cd/m^2^) at a viewing distance of 10–25 cm. All stimuli were presented in a circular aperture surrounded by a gray field of mean luminance. Orientation selectivity and surround suppression were characterized online using drifting square wave gratings. Spatiotemporal tuning was then assessed with full contrast drifting sine wave gratings with 36 combinations of SFs [0.01, 0.02, 0.04, 0.08, 0.16 and 0.32 cycles per degree (cpd)] and TFs (0.25, 0.5, 1, 2, 4, 8 Hz). All spatiotemporal and adapting stimuli were presented at the optimal orientation and size for each unit, and drifted in the direction that elicited maximal excitation. Presentations of each combination of SF and TF were randomized with 8–10 repeats for each stimulus. The presentation time of the stimulus was 1.5 s, and a gray of mean luminance was shown between stimuli for 0.5 s. Grating start-phase was staggered on each repetition to average out periodic firing of phase-sensitive neurons. The spatiotemporal tuning of each unit was then examined online and appropriate adaptors were selected for the subsequently presented contrast adaptation protocols. Two stimulus protocols that have previously been used to investigate contrast adaptation in mice, cats, and primates were modified to study the spatiotemporal specificity of contrast adaptation in mouse V1: top-up adaptation (Sclar et al., 1989; Duong and Freeman, 2007; Stroud et al., 2012), and contrast ramps (Crowder et al., 2008; Stroud et al., 2012).

#### Top-up adaptation

We chose the top-up contrast adaptation protocol because it has commonly been used to study the stimulus specificity of contrast adaptation in higher mammals (e.g., Movshon and Lennie, 1979; Duong and Freeman, 2007; Dhruv et al., 2011), which facilitates cross-species comparisons. Sine-wave contrast is defined as:

(1) $$\text{Michelson contrast}=\frac{\left({\text{Luminance}}_{\text{max}}\;-\;{\text{Luminance}}_{\text{min}}\right)}{\left({\text{Luminance}}_{\text{max}}\;+\;{\text{Luminance}}_{\text{min}}\right)}$$

where Luminance_max_ and Luminance_min_ are the maximum and minimum luminances, respectively. Non-adapted contrast response functions were obtained by recording responses to ten contrasts (0.04, 0.08, 0.12, 0.16, 0.24, 0.32, 0.48, 0.64, 0.82, 1) presented in random order for 0.5 s tests (8–12 repetitions) interleaved with 4 s of mean luminance. Adapted contrast response functions were collected in blocks where: (1) the adapting grating matched the cell's spatiotemporal peak; (2) the SF of the adapting grating was 1–3 octaves higher or lower than the cell's preferred SF; and (3) the TF of the adapting grating was set to 8 Hz. Adaptation blocks consisted of 60 s of the adapting grating at a contrast of 0.32 followed by 0.5 s tests (aforementioned contrasts for 8–12 repetitions) interleaved with 4 s adaptation top-ups. An adapting contrast of 0.32 was chosen because our previous study of contrast adaptation in mouse V1 (Stroud et al., 2012) indicated that this contrast produced reliable adaptation while still allowing the data to be easily fit with sigmoid curves (see Curve Fitting below).

#### Contrast ramps

One drawback of the top-up protocol described above is that it takes a long time to record even a single adapted contrast response function (Sclar et al., 1989; Crowder et al., 2006). Therefore, when exploring the SF or TF specificity of contrast adaptation, only a few conditions can be examined for any single cell. To more fully assess the nature of contrast adaptation in the spatiotemporal domain, we used contrast ramp stimuli. Contrast ramps are dynamic contrast stimuli where the contrast of the sine wave grating is changed linearly on each animation frame over the time-course of the presentation. Importantly, these ramps are able to measure several key markers of contrast adaptation with fairly short presentation times (Crowder et al., 2008; Stroud et al., 2012). Contrast ramp stimuli were first presented at a contrast of 0, and contrast was increased linearly over 2 s until it reached 1 (rising phase). The contrast of the grating was then ramped back down from 1 to 0 (falling phase) over the next 2 s. Thus, the neuron is presented with identical contrasts in the rising and falling phases, but the order of presentation (i.e., temporal context) is reversed. A full screen gray of mean luminance was shown between ramp stimuli for 2 s. In this protocol, the spatiotemporal specificity of contrast adaptation was tested by varying the SF and TF of the contrast ramps using the 36 combinations of SFs (0.01, 0.02, 0.04, 0.08, 0.16, and 0.32 cpd) and TFs (0.25, 0.5, 1, 2, 4, 8 Hz) that were directly comparable with the spatiotemporal profile obtained for each neuron. Contrast ramps with different spatiotemporal combinations were randomized and repeated 8–12 times for each combination.

We were interested in determining whether the spatiotemporal combination that caused maximal firing also caused maximum hysteresis between the rising and falling portions of the contrast ramp. In order to test this, we used a *symmetrical* contrast ramp procedure, which maintained the same spatiotemporal parameters for both the rising and falling phase of the ramp. We also collected a second type of contrast ramp from a subset of neurons referred to as *peak-tested* contrast ramps that were more directly comparable to the top-up protocol. In the peak-tested protocol, the rising phase of the contrast ramp was one of the 36 combinations of SF and TF, but the falling phase was always shown at the neuron's preferred SF and TF (see Results), as chosen from the online tuning function.

### Initial data analysis

Spike sorting was performed offline with Spike2 software, which first searched for and sorted spikes using a supervised template-matching algorithm, and then displayed candidate spikes with a principle components analysis for approval. Data was exported to MatLab and neuronal responses were represented as spike density functions (SDF) with 1 kHz resolution generated by convolving a delta function at each spike arrival time with a Gaussian window. For each unit, we calculated the magnitude of orientation, size, and spatiotemporal tuning using a discrimination index (DI) (DeAngelis and Uka, 2003):

(2) $$\text{DI}=\frac{\left({\text{Resp}}_{\text{Max}}-{\text{Resp}}_{\text{Min}}\right)}{\left(\left({\text{Resp}}_{\text{Max}}-{\text{Resp}}_{\text{Min}}\right)+\text{2}\sqrt{\text{SSE}}/(N-M)\right)}$$

Resp_Max_ is the neuron's max response, while Resp_Min_ is the neuron's minimum response. SSE is the sum of squared error of the mean, *N* is the total number of presentations of the stimuli, and M is the number of different stimuli presented. In order to classify cells as simple or complex, we divided the first Fourier coefficient of a neuron's response to a grating near the spatiotemporal peak (*F*_1_) by the mean time-averaged response to this grating (*F*_0_) (Movshon et al., 1978a,b; Skottun et al., 1991). Despite some recent controversy (Mechler and Ringach, 2002; Crowder et al., 2007; Henry and Hawken, 2013; Hietanen et al., 2013), the *F*_1_/*F*_0_ ratio has been used to quantitatively classify simple and complex cells in numerous studies, and an *F*_1_/*F*_0_ ratio less than 1 indicates a cell is complex.

#### Curve fitting

We used the least squares method to fit contrast response functions. Sigmoid curves (Albrecht and Hamilton, 1982) were fit to the mean responses from top-up contrast response functions and SDFs produced by contrast ramps:

(3) $$\text{R}\left(c_{i}\right)=\frac{R_{\max}\times c_{i}^{n}}{c_{i}^{n}+c_{50}^{n}}+\text{M}$$

where R(*c_i_*) is the amplitude of the evoked response at contrast *c_i_*, M is the spontaneous rate, *n* is the exponent that determines the steepness of the curve, R_max_ is the maximum elevation in response above the spontaneous rate, and c_50_ is the contrast that generates a response elevation of half R_max_. Response saturation was evident for almost all non-adapted top-up contrast response functions and rising ramp responses allowing for well constrained fits. When fitting adapted curves where the response to maximal contrast was similar to or less than the non-adapted response but saturation was not evident, we assigned an upper bound on the adapted R_max_ of 15% above the non-adapted R_max_ in order to obtain tractable fits.

#### Neuronal latency

To examine the amount of hysteresis for each contrast ramp, responses were latency-corrected as previously described (Crowder et al., 2008; Stroud et al., 2012). Briefly, for each unit a response threshold was established based on the 99% cut-off from a Poisson distribution fitted to the spontaneous firing rate. Each unit's response latency was calculated as the first time the spiking rate in the response to gratings of optimal SF and TF (from the spatiotemporal tuning stimulus) exceeded the aforementioned Poisson threshold and stayed above the threshold for the subsequent 25 ms (Price et al., 2005). For each unit, responses to contrast ramps were shifted back in time by the neural latency then split into the rising and falling phases and re-plotted using units of contrast on the abscissa instead of time (which resulted in the falling phases of contrast ramps being flipped left-to-right).

## Results

**Figure 1:**
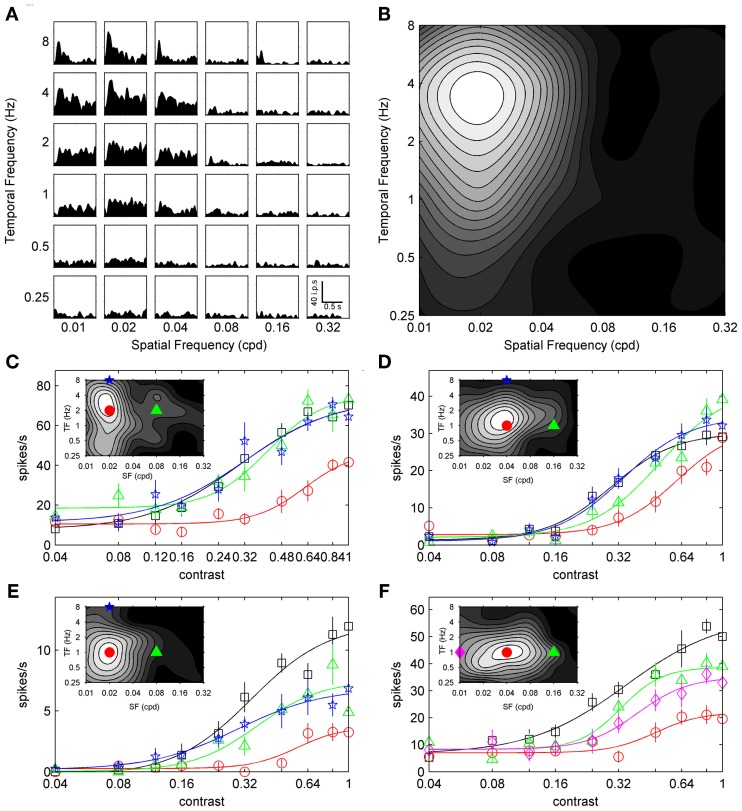
**Spatiotemporal specificity of top-up contrast adaptation.** The spatiotemporal selectivity of a sample neuron is presented in **(A)**, with a grid of SDFs showing the neuron's response to gratings with different combinations of SF (columns) and TF (rows). A scale bar depicting time vs. impulses per second (ips) is shown in the bottom right SDF (0.32 cpd and 0.25 Hz). Mean responses from SDFs are summarized as a grayscale contour plot in **(B)** (maximal firing shown in white), with SF on the abscissa and TF ordinate. **(C–F)** show contrast response functions from four sample neurons with contrast on the abscissa and mean response rate on the ordinate. Non-adapted, preferred adapted, non-preferred SF adapted, and non-preferred TF adapted responses are shown as black squares, red circles, green triangles, and blue pentagrams, respectively. **(F)** shows responses following an additional non-preferred SF adaptor as pink diamonds. Error bars represent SEM, and thin lines represent best fits to a sigmoid function (see Materials and Methods). Spatiotemporal tuning of each sample neuron is shown in the corresponding inset with adapting stimulus SF and TF indicated with symbols matched to contrast response functions.

Recordings were collected from 188 visually responsive units in the primary visual cortex of 25 C57BL/6 J mice. We obtained contrast adaptation data from 65 units using the top-up protocol, and 125 units using the ramp protocol (*n* = 90 for *symmetrical* contrast ramps; *n* = 35 for *peak-tested* contrast ramps). The stimulus preferences of units in our sample were generally consistent with previous reports. Discrimination indices for orientation selectivity (0.49 ± 0.1; mean ± s.d.), size tuning (0.62 ± 0.1), and spatiotemporal selectivity (0.64 ± 0.08) were similar to those reported by Gao et al. (2010). Peak SFs and TFs were broadly distributed, with preferred SFs ranging from 0.01 to 0.18 cpd (mean = 0.03 cpd) and preferred TFs ranging from 0.25 to 8 Hz (mean = 1.77 Hz). Figure 1A shows the grid-like array of responses used to measure the spatiotemporal tuning of a sample neuron, and Figure 1B shows how these responses can be summarized as a contour plot to indicate the combination of SF and TF that produced the maximal response. Our range of peak SFs and TFs were similar to recent electrophysiological studies of mouse visual cortex (Niell and Stryker, 2008; Gao et al., 2010; LeDue et al., 2012), and within the ranges shown by recent multi-photon calcium imaging studies (Andermann et al., 2011; Marshel et al., 2011). Finally, 157 units were classified as complex (*F*_1_/*F*_0_ ratio < 1) and 35 units were classified as simple (*F*_1_/*F*_0_ ratio > 1). Since simple and complex cells showed similar trends for all measures of contrast adaptation, they were pooled into a single group.

### Top-up contrast adaptation

Robust contrast adaptation following prolonged exposure to an adaptor of the preferred SF and TF has been shown previously in mouse V1 (Stroud et al., 2012). However, to our knowledge the spatial and temporal frequency specificity of contrast adaptation in mouse V1 have not been explored. Therefore, we compared the magnitude of contrast adaptation induced by an adaptor with preferred SF and TF with that induced by an adaptor with non-preferred SF or TF. We chose a non-preferred adapting TF of 8 Hz because high TFs rarely elicited strong responses. This high TF adaptor also permitted comparisons with primate work, which has shown that high TFs can reliably induce contrast adaptation in V1 without strongly driving the recorded neurons (Dhruv et al., 2011), presumably by inducing adaptation in the LGN (Solomon et al., 2004). We selected non-preferred SFs 1–3 octaves higher or lower than the peak SF depending on the breadth and location of the recorded unit's spatiotemporal tuning. Care was taken to ensure non-preferred SFs elicited weak responses from the recorded unit but also were within the range of peak SFs of our sample population and below the mean SF cutoff reported in previous studies of LGN and V1 (Grubb and Thompson, 2003; Gao et al., 2010). Higher adapting SFs were selected more often than lower ones since SFs lower than 0.01 cpd can begin to appear as global changes in luminance within the stimulus aperture.

Figures 1C–F shows the SF and TF specificity of contrast adaptation for four example neurons. Contrast response functions are shown for non-adapted (black squares), preferred adapted (red circles), non-preferred SF adapted (green triangles), and non-preferred TF adapted (blue stars) conditions. For the cell in Figure 1F, contrast response functions from two different non-preferred SFs (low SF = pink diamonds; high SF = green triangles) are shown. The spatiotemporal tuning of each unit is shown inset with the SF and TF values of the adapting stimuli indicated with matching symbols. In each case the preferred adaptor induced the most contrast adaptation. Non-preferred adaptors either induced virtually no adaptation (Figures 1C,D), or less adaptation than the preferred stimulus (Figures 1E,F).

**Figure 2:**
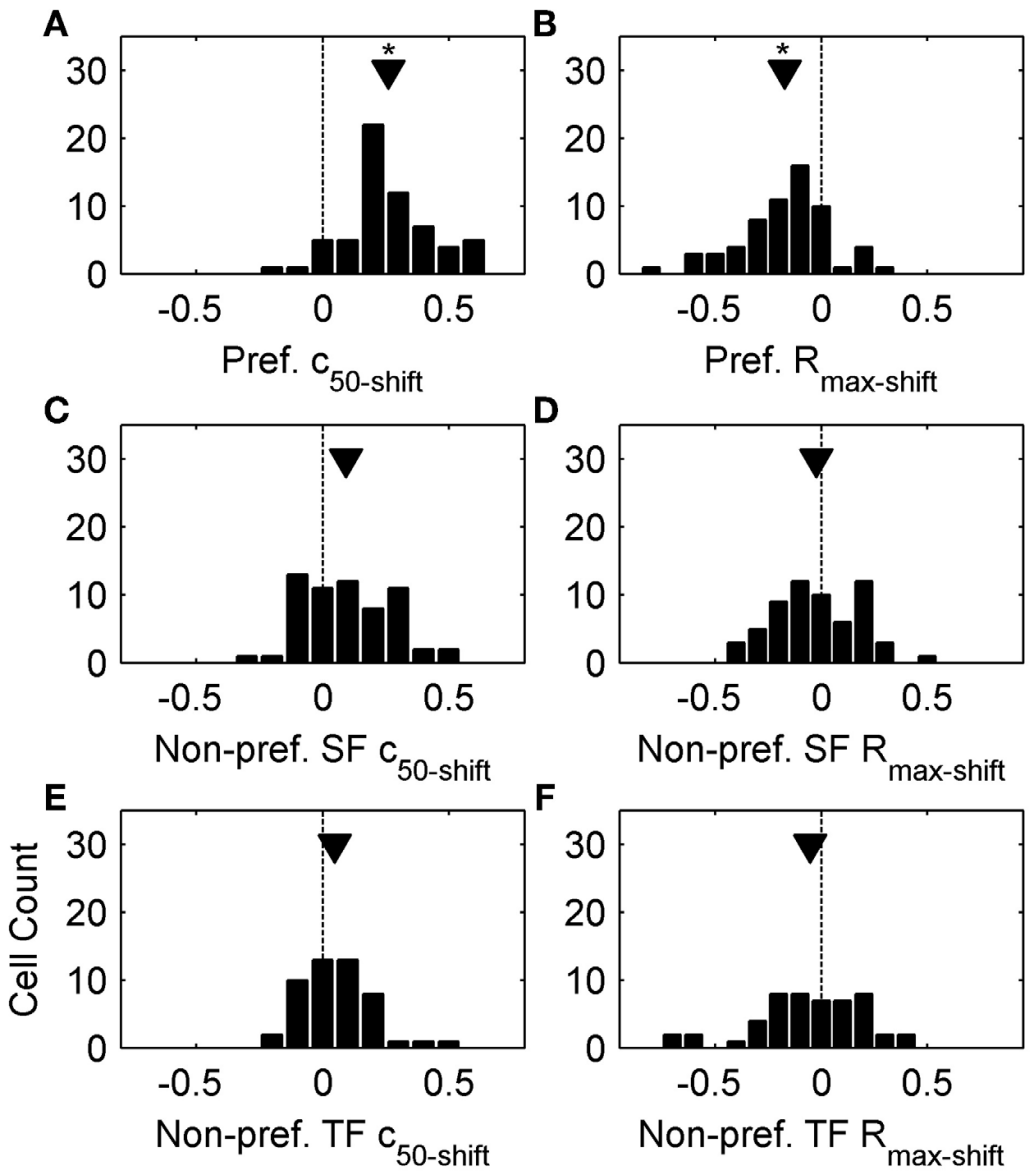
**Population data from top-up adaptation. (A,C,E)** plot c_50-shift_ population histograms following preferred adaptation, adaptation with non-preferred SFs, and adaptation with non-preferred TFs, respectively. **(B,D,F)** plot R_max-shift_ population histograms following preferred adaptation, adaptation with non-preferred SFs, and non-preferred TFs, respectively. Arrowheads represent population means, and asterisks denote significant differences (see Results).

Sigmoid fits to each contrast response function are shown as thin lines in Figure 1, and we used the c_50_ and R_max_ parameters extracted from these fits to quantitatively analyze changes in contrast response functions following top-up adaptation. For each adaptation condition we measured the change from the non-adapted curve as a difference-over-sum calculation (parameter_shift_ = [adapted – non-adapted]/[adapted + non-adapted]), and plotted this metric as population histograms in Figure 2. For Figures 2A,C,E positive values of c_50-shift_ indicate a rightward shift in the adapted contrast response function. Nearly all cells showed a rightward shift following preferred adaptation (Figure 2A, mean c_50-shift_ = 0.26), but the population was centered closer to zero for both adaptors with non-preferred SF (Figure 2C, mean c_50-shift_ = 0.09) and TF (Figure 2E, mean c_50-shift_ = 0.05). A One-Way ANOVA followed by a Tukey-Kramer *post-hoc* indicated that the preferred adaptation produced significantly larger values of c_50-shift_than the other two adaptation conditions [*F*_(2, 169)_ = 27.54, *p* < 0.001], while non-preferred SF and TF c_50-shift_ did not differ. For Figures 2B,D,F negative values of R_max-shift_ indicate a decrease in firing to maximal contrast following adaptation. Most cells showed a modest decrease in R_max_ following preferred adaptation (Figure 2B, mean R_max-shift_ = −0.17), but the population was centered near zero for both adaptors with non-preferred SF (Figure 2D, mean R_max-shift_ = 0.02) and TF (Figure 2F, mean R_max-shift_ = 0.05). A One-Way ANOVA followed by a Tukey-Kramer *post-hoc* showed similar results to the c_50_ data, with preferred adaptation producing significantly more negative values of R_max-shift_ than the other 2 adaptation conditions [*F*_(2, 169)_ = 7.72, *p* < 0.001], while non-preferred SF and TF R_max-shift_ did not differ. Another way of quantifying the spatiotemporal specificity of contrast adaptation is simply to rank order the adapted curves for each cell. Preferred adaptation c_50_ values were larger than c_50_ values measured following non-preferred SF adaptation for 90% of cells, and non-preferred TF adaptation for 92% of cells. Preferred adaptation R_max_ values were smaller than R_max_ values measured following non-preferred SF adaptation for 75% of cells, and non-preferred TF adaptation for 70% of cells.

### Contrast ramp adaptation

**Figure 3:**
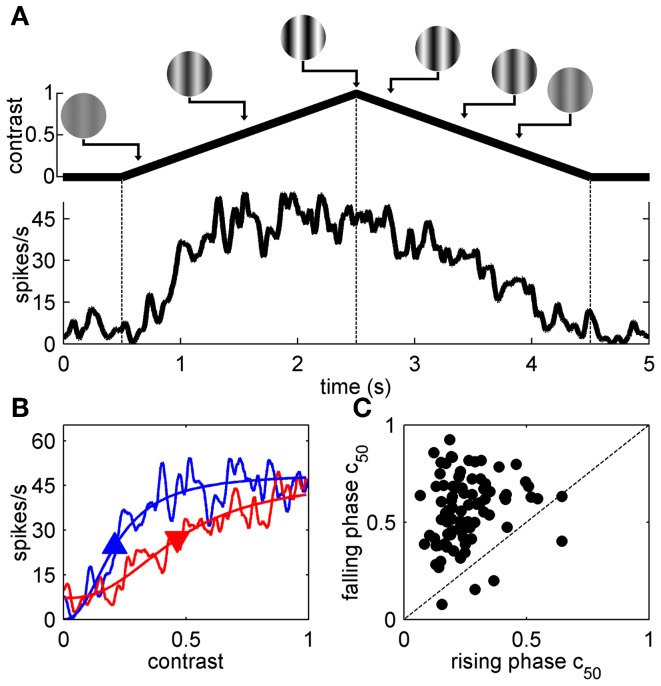
**Contrast ramps of optimal SF and TF. (A)** shows the response of a representative neuron to a contrast ramp of optimal SF and TF along with a schematic demarcating the rising and falling phases of the contrast ramp with dashed lines. In **(B)**, the neuron's responses to the rising (blue lines) and falling phases (red lines) of the contrast ramp are compared by folding the latency-corrected SDF back on itself so that contrast is on the abscissa and spikes/s is on the ordinate (see Materials and Methods). SDFs were fit with sigmoid functions (thin lines), and upward and downward pointing arrowheads represent c_50_ values obtained from fits to the rising and falling phases, respectively. Population data comparing c_50_ values obtained from fits to the rising (abscissa) and falling phase responses (ordinate) is shown in **(C)**.

The top-up adaptation data above clearly demonstrates that the magnitude of contrast adaptation in mouse V1 depends on the adapting SF and TF, however, as noted in the Methods section only a few adaptation conditions can be studied for any single cell due to the time constraints imposed by this protocol. Therefore, we used *symmetrical* contrast ramp stimuli to more extensively map the spatiotemporal selectivity of contrast adaptation. Figure 3A shows the response of a representative neuron to a contrast ramp of optimal SF and TF. Even though the rising and falling phases of the ramp stimulus are symmetrical, the spiking response shows clear hysteresis. If this spiking response is latency-corrected and re-plotted with contrast on the abscissa (see Materials and Methods), the difference between the responses to the rising (red) and falling (blue) phases of the contrast ramp is accentuated further (Figure 3B). As in previous studies (Crowder et al., 2008; Stroud et al., 2012), the SDFs were fit to sigmoid curves (thin lines). The most useful parameter extracted from the sigmoid fits was c_50_, since it captured the rightward shift in the contrast response function by comparing semi-saturation contrasts of the rising (upward pointing arrowhead) and falling phases (downward pointing arrowhead) of the contrast ramp. For our sample (*n* = 90), c_50_ values from the rising phase were almost always smaller than c_50_s from the falling phase (Figure 3C), and this difference was significant (*p* < 0.01, paired *t*-test). This replicates earlier findings in cats (Crowder et al., 2008), and mice (Stroud et al., 2012).

**Figure 4:**
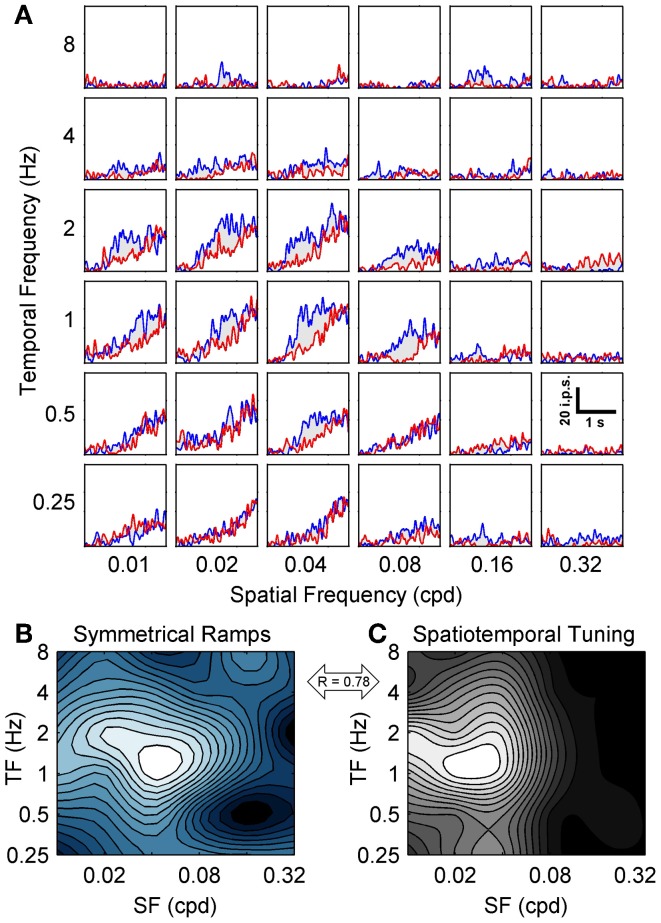
**Spatiotemporal specificity of contrast ramp adaptation.** The grid of SDFs in **(A)** show the hysteresis induced in a sample neuron by symmetrical contrast ramps of varying TFs (rows) and SFs (columns). Each SDF follows a similar format to Figure 3B, with responses to the rising phase of the contrast ramp shown in blue and responses to the falling phase shown in red. A scale bar depicting time vs. impulses per second (ips) is shown in the lower right (0.32 cpd and 0.5 Hz). The spatiotemporal pattern of hysteresis for the sample neuron is represented as a blue-tinted contour plot in **(B)**, with larger mean differences between the rising and falling phase responses shown as more desaturated hues (see Results). For comparison, the spatiotemporal tuning of the sample neuron is shown in **(C)** as a grayscale contour plot, and the correlation between the two contour plots is indicated (double-headed arrow). For both contour plots SF is on the abscissa and TF in on the ordinate.

**Figure 5:**
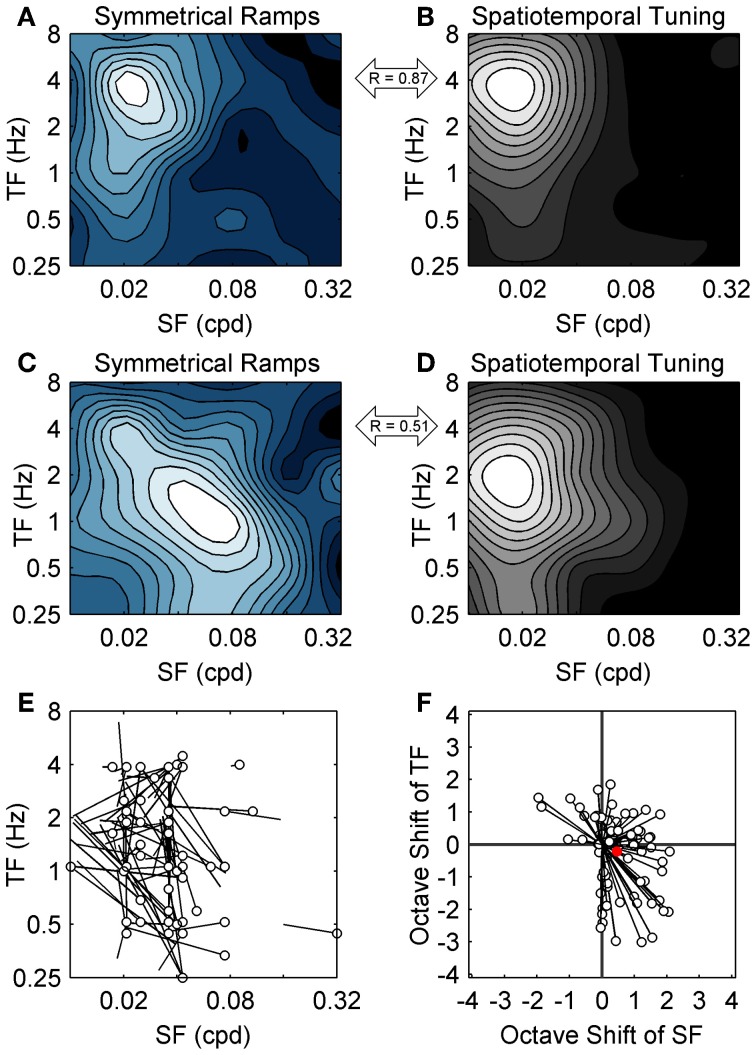
**Population data from symmetrical contrast ramp adaptation**. The spatiotemporal specificity of adaptation induced with contrast ramps (left column) and spatiotemporal tuning (right column) are compared for two additional sample neurons. As in Figure 4, contrast ramp hysteresis is shown as blue-tinted contour plots, and spatiotemporal tuning is shown as grayscale contour plots. The contour plots from the neuron in the top row **(A,B)** match quite closely, indicating that similar grating parameters produced maximum firing and maximum contrast ramp hysteresis. The contour plots from the second neuron (middle row; **C,D)** match less closely, with the spatiotemporal location of maximum hysteresis occurring at a higher SF and lower TF than the peak in spatiotemporal tuning. The correlations between the contrast ramp and spatiotemporal tuning contour plots are indicated for each neuron (double-headed arrows). **(E)** shows population data comparing the spatiotemporal location of maximal hysteresis (empty dots) with the locations of peak responding from spatiotemporal tuning (lines). For **(A–E)**, SF is on the abscissa and TF in on the ordinate. **(F)** normalized the data from **(E)** by calculating octave differences in SF (abscissa) and TF (ordinate) to show the location of maximal contrast hysteresis (empty dots) relative to each cell's spatiotemporal tuning (all normalized to 0). Population mean is shown as a solid red circle.

To map the spatiotemporal specificity of contrast adaptation we measured the hysteresis of ramp responses when the SF and TF of the ramp grating were varied (for easy comparison to the spatiotemporal tuning also obtained for each neuron we used the same 36 combinations of SF and TF). This stimulus protocol examined the spatiotemporal specificity of contrast adaptation from a slightly different perspective than the top-up protocol. The top-up protocol measured whether the magnitude of contrast adaptation was affected if the adapting grating did not match the test grating, which emphasized the importance of the adapting stimulus. Symmetrical contrast ramps measured the combination of SF and TF that produced the most hysteresis, which emphasized the importance of the cell's own preferred stimulus in determining the strength and specificity of the adaptation effect (Saul and Cynader, 1989a). Figure 4A shows a grid of SDF ramp responses from a sample cell, each with the same format as Figure 3B. This neuron had strong ramp responses with substantial hysteresis around 0.02–0.04 cpd and 1–2 Hz. Responses to lower TFs (~0.25 Hz) showed little hysteresis despite monotonic increases in firing with contrast, and the entire ramp response flattened out at the highest SFs and TFs. The former effect was observed in 81/90 neurons, indicating that diminished adaptation was not solely due to lack of responding. We wanted to summarize the pattern of hysteresis for each neuron as a contour plot, but c_50_ values taken from sigmoid fits to SDFs were unreliable for spatiotemporal combinations away from the peak, so we measured adaptation by calculating the mean difference between the responses to rising and falling phases of the contrast ramps. During adaptation, the semi-saturation contrast of the falling phase ramp response shifts to higher values, causing the falling ramp response to be lower than the rising phase response at most contrasts. This method of analysis has been shown by Stroud et al. (2012) to capture the major features of contrast adaptation without relying on fitting the ramp SDFs to sigmoid functions. Figure 4B shows the contour plot for this neuron summarizing the magnitude of hysteresis evoked by each combination of SF and TF. The first feature to note is the clear peak around 0.04 cpd and 1–2 Hz. Likewise, 85 out of 90 units produced contour plots with an easily identifiable single peak that was at least four times higher than the level of hysteresis produced by the least effective ramp. This supports our earlier finding of spatiotemporal specificity of contrast adaptation using a different method. The second feature of the hysteresis contour plot that we were interested in was whether the combination of SF and TF that produced maximum hysteresis for contrast ramps matched the neuron's peak in the spatiotemporal domain tested with regular grating blocks (Figure 4C). For this neuron, the two contour plots look similar (*R* = 0.78 from a 2D correlation analysis), but the gratings that produced maximum hysteresis had a slightly higher SF than the gratings that produced maximum firing. Figure 5 shows two more example cells, one where the spatiotemporal locations of maximum firing and maximum hysteresis match quite closely (Figures 5A,B; *R* = 0.87), and another where spatiotemporal location of maximum hysteresis is at a higher SF and lower TF (Figures 5C,D; *R* = 0.51). Figure 5E plots the difference in peak locations from the two types of contour plots in the spatiotemporal domain for each cell as “hatpins” (*n* = 85), with the empty dots indicating the location of maximum hysteresis. No pattern was apparent, indicating that there was not one specific combination of SF and TF that universally induced maximal hysteresis across cells. Figure 5F normalized the data from Figure 5E by calculating the octave difference in SF and TF between each pair of peaks to show the location of maximal contrast hysteresis (empty dots) relative to each cell's peak in spatiotemporal tuning (all normalized to 0). Although the differences were small (45% of cells had peaks within 1 octave of each other, and the median *R*-value from 2D correlations was 0.71), at the population level the gratings that produced maximum hysteresis tended to have slightly higher SFs (mean: 0.47 octaves) and lower TFs (mean = − 0.22 octaves) than the gratings that produced maximum firing. A 2-way repeated measures ANOVA (with peaks from spatiotemporal tuning vs. hysteresis contour plots and SF vs. TF as factors) showed a significant main effect of peak type [*F*_(1, 84)_ = 3.95, *p* < 0.05], indicating that the spatiotemporal location of peak hysteresis and peak firing tended to be different. Furthermore, a significant interaction between factors indicated that the difference between peaks was larger for SF than for TF [*F*_(1, 84)_ = 15.75, *p* < 0.001].

**Figure 6:**
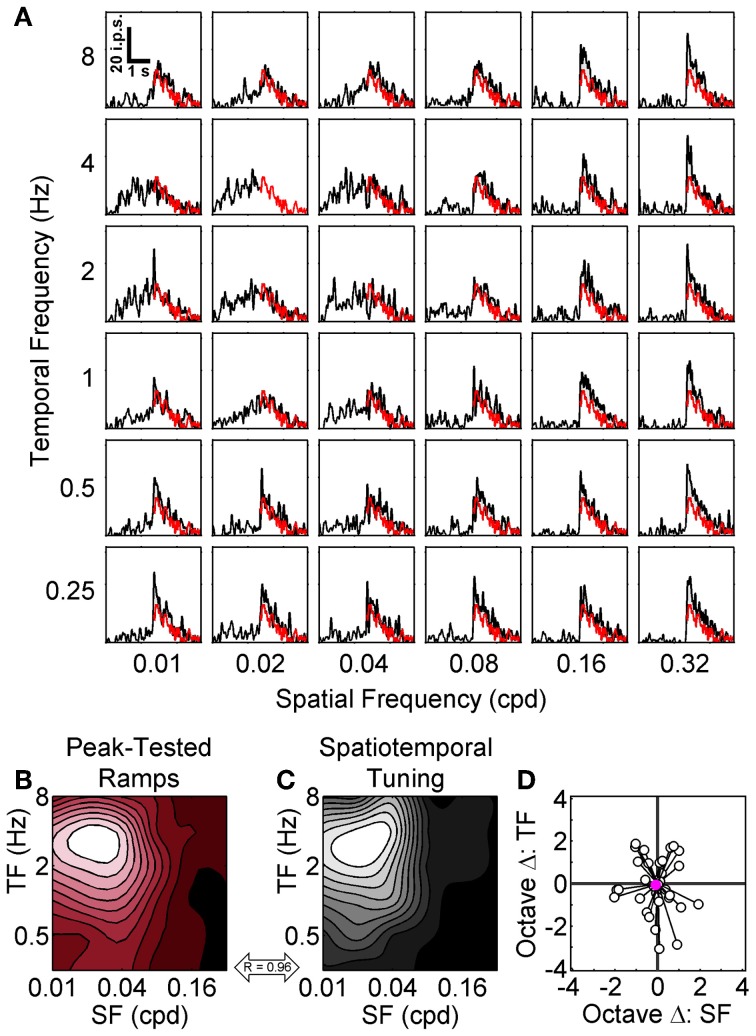
**Peak-tested contrast ramp adaptation.** The grid of SDFs in **(A)** show the responses of a sample neuron to peak-tested ramps where the TFs (rows) and SFs (columns) of the rising phase of the ramp were varied, but the falling phase was always shown at the neuron's peak SF and TF (red lines). The transition between non-preferred and preferred gratings is especially apparent at high SFs. A scale bar depicting time vs. impulses per second (ips) is shown in the top left (0.01 cpd and 8 Hz). The spatiotemporal pattern of adaptation for the sample neuron is represented as a red-tinted contour plot in **(B)**, with smaller mean differences between the preferred falling phase and other falling phase responses shown as more desaturated hues (see Results). For comparison, the spatiotemporal tuning of the sample neuron is shown in **(C)** as a grayscale contour plot, and the correlation between the two contour plots is indicated (double-headed arrow). For both contour plots SF is on the abscissa and TF in on the ordinate. **(D)** shows the octave differences in SF (abscissa) and TF (ordinate) between the locations of maximal contrast adaptation (empty dots) relative to each cell's spatiotemporal tuning (all normalized to 0). Population mean is shown as a solid pink circle.

Considering that we consistently observed the strongest adaptation when using the preferred SF and TF in the top-up protocol we were surprised by the results of the ramp protocol. However, as noted above there was one key difference between adapting procedures: in the top-up protocol the adapting grating varied but the test gratings were always set at the preferred SF and TF, whereas in the symmetrical ramp protocol the SF and TF of the grating remained constant throughout the rising and falling phases of the contrast ramp. To determine whether the different adaptation effects observed between the top-up and contrast ramp protocols were due to switching the SF/TF between adapting and test stimuli or the dynamic nature of the contrast ramp stimuli we presented a subset of cells with a modified ramp protocol referred to as *peak-tested* ramps. For these peak-tested ramps, the rising phase could have any one of the 36 combinations of spatiotemporal frequencies (a proxy for the adapting gratings in the top-up protocol), but the falling phase was always shown at the neuron's preferred spatiotemporal frequency (a proxy for the test gratings). For these stimuli, we compared the response to the falling phase at the spatiotemporal peak with the falling phase responses at every other spatiotemporal combination since these stimuli were identical with only the preceding rising phase differing (Figure 6A). We expected the difference to be large if no contrast adaptation occurred, or small if contrast adaptation did occur. Responses from a representative neuron are shown in Figures 6A–C. We again represented the spatiotemporal specificity of adaptation for each neuron as a contour plot (Figure 6B), compared the adaptation contour plot to each neuron's spatiotemporal profile (Figure 6C), and calculated the octave difference in SF and TF between peaks for the population (Figure 6D). For peak-tested ramps, there were clear peaks in the hysteresis contour plots of every cell (e.g., Figure 6B). Importantly, the contrast hysteresis and spatiotemporal profile contour plots were much more similar using this protocol. For the sample neuron shown in Figures 6A–C the 2D correlation between contour plots was 0.96, and the population median was 0.86. Furthermore, the 2D correlations between contour plots for the peak-tested protocol were significantly higher than for the symmetrical ramp protocol (*p* < 0.0001; *t*-test). Mean octave differences in peak location between contour plots were −0.08 and −0.06 for SF and TF, respectively (Figure 6D). A Two-Way repeated measures ANOVA (with peaks from spatiotemporal tuning vs. hysteresis contour plots and SF vs. TF as factors) indicated that neither the main effect of peak type [*F*_(1, 34)_ = 0.31, *p* > 0.57], nor the interaction between factors were significant [*F*_(1, 34)_ = 0.01, *p* > 0.92]. Thus, switching the SF/TF between adapting and test stimuli appear to be the important difference between the top-up and symmetrical ramp protocols because when the contrast ramp stimulus was altered to more closely resemble the top-up protocol the adaptation effects also matched the top-up results more closely. Overall, each of the three adaptation protocols along with their differing methods of analysis demonstrated the spatiotemporal specificity of contrast adaptation in mouse V1, even though differences between protocols produced some subtle variations in the nature of the adaptation.

## Discussion

This study demonstrated that contrast adaptation in mouse V1 is specific in the spatiotemporal domain. The magnitude of contrast adaptation observed in single units was found to depend on both the SF and TF of the adapting grating, and the nature of the adaptation effect could also be affected by the SF and TF of the test stimuli. The properties of contrast adaptation we observed were broadly similar to single unit studies in higher mammals (monkeys: Sclar et al., 1989; Dhruv et al., 2011; cats: Movshon and Lennie, 1979; Ohzawa et al., 1982, 1985; Saul and Cynader, 1989a,b; Bonds, 1991) and psychophysical data (e.g., Blakemore and Campbell, 1969). This suggests that contrast adaptation can be thought of as a general feature of the mammalian geniculo-striate pathway along with other classical response properties (e.g., Niell and Stryker, 2008; Gao et al., 2010; Van den Bergh et al., 2010). Despite marked differences between animal models (frontal eyes vs. lateral eyes, nocturnal vs. diurnal, acuity that varies over several orders of magnitude), adaptation in mouse visual cortex appears to follow similar rules and is of similar complexity to higher mammals. We believe that these findings uphold the viability of the mouse model for studying vision, and support the validity of a multi-species approach for investigating cortical visual processing.

### Comparison with previous studies

To our knowledge only two previous studies have investigated contrast adaptation in mouse V1 (Niell and Stryker, 2008; Stroud et al., 2012). Stroud et al. (2012) were able to make direct comparisons between adaptation in mouse and cat V1 neurons, and reported that most key features of contrast adaptation were similar between species. When adapted and tested with an optimal grating, adaptation shifted contrast response functions down and to the right. Moreover, contrast ramps produced relatively robust contrast adaptation given their brief presentation times. The current study is in agreement with these previous findings, so the Discussion will focus on the spatiotemporal specificity of contrast adaptation.

Several previous papers have used some version of the top-up protocol to investigate either the SF or TF dependence of contrast adaptation (e.g., Movshon and Lennie, 1979; Duong and Freeman, 2007; Dhruv et al., 2011), and are therefore readily comparable to our own top-up data. Within our top-up protocol, the test stimuli were always at the optimal SF and TF for each neuron, which is most similar to the stimuli used by Dhruv et al. (2011) in their study of TF and orientation specificity of contrast adaptation in macaque V1. For these two studies, one central question was whether an adapting stimulus that itself does not strongly drive the recorded neuron could induce adaptation. Another study examining the SF specificity of contrast adaptation in cat V1 used a complementary design where the test gratings were not at each neuron's optimal SF, but rather at an SF that evoked approximately the same firing rate as the adapting grating (Movshon and Lennie, 1979). Regardless of these design differences, the general findings of these studies indicate that contrast adaptation is most robust when the parameters of the adapting grating are similar to the test grating. The current study extends this finding into a genetically tractable animal model where there exists an expanded toolbox to investigate the mechanisms underlying this specificity.

Comparing orientation specificity and spatiotemporal specificity of contrast adaptation in mouse V1 is also worthwhile. Most neurons in V1 adapt to any orientation, even ones that elicit low firing rates (Stroud et al., 2012), which contrasts with our current results in the spatiotemporal domain. This pattern of results could be produced if cortical adaptation mechanisms pooled over orientation (or the sharpness of tuning was diluted by non-oriented cells), but were at least somewhat selective in the spatiotemporal domain (Andermann et al., 2011; LeDue et al., 2012).

It has been shown that adapting gratings with a high TF can induce modest but reliable contrast adaptation in macaque V1 without strongly driving the recorded neuron (Dhruv et al., 2011), presumably by inducing adaptation in magnocellular cells in the LGN (Solomon et al., 2004). Therefore, we were somewhat surprised to observe only occasional adaptation to higher TFs in our data set. In their study of macaque V1, Dhruv et al. (2011) used an adaptor with a TF of 30–50 Hz, which was 2–3 times higher than the peak TF of LGN neurons (10–16 Hz: Derrington and Lennie, 1984; Hawken et al., 1996). For our top-up protocol, the high TF adaptor (8 Hz) was also approximately double the peak TF of mouse LGN neurons (3.8 Hz: Grubb and Thompson, 2003), yet we observed little consistent adaptation to this stimulus. We had also predicted that the peaks in the contrast hysteresis contour plots obtained with our contrast ramp stimuli may be skewed toward higher TFs, but this was not the case. We are unsure what underlies this apparent species difference, but as outlined in a model of multiple sources of adaptation used by Dhruv et al. (2011), it suggests that less adaptation is occurring in (or being inherited from) the LGN in mice. This observation, in conjunction with the finding that contrast adaptation in cat LGN does not show SF specificity (Duong and Freeman, 2007), provide two good reasons for future work to investigate adaptation in mouse LGN.

Finally, the contrast ramp stimuli we used in this study were quite unique and therefore less comparable to previous papers (although the staircase-like stimuli used by Bonds (1991) also measured hysteresis when contrasts were presented in an ordered manner). However, this data is relevant to a longstanding issue in the contrast adaptation literature. Vautin and Berkley (1977) were the first to discuss how the adaptation measured in a recorded neuron could arise from processes occurring within the cell itself (intrinsic) or be inherited from other neurons in the circuit/network (extrinsic). Saul and Cynader (1989a) suggested that intrinsic mechanisms may be more narrowly tuned since they depend on the cell's own tuning, while the aforementioned model by Dhruv et al. (2011) specified that some extrinsic sources of adaptation should be broadly tuned for certain stimulus attributes like orientation. The main strength of the contrast ramp stimulus is that it can measure features of contrast adaptation on a relatively short time-scale, and this allows for a large stimulus-space to be explored in a reasonable amount of time. This seems ideal for exploring the putative differences in tuning between intrinsic and extrinsic sources of adaptation. In the current study, both the symmetrical and peak-tested contrast ramp protocols support the spatiotemporal specificity of contrast adaptation initially described using the top-up protocol despite the fact ramp stimuli measured adaptation on a different time scale and used different metrics. It would be interesting to obtain comparative data from higher mammals for these stimuli.

### Using mouse models to study contrast adaptation

Electrophysiological studies in various animal models additively suggest that contrast adaptation is initiated at pre-cortical stages (retina: reviewed in Demb, 2008; LGN: Sanches-Vives et al., 2000a; Solomon et al., 2004; Duong and Freeman, 2007), and then refined and strengthened in the cortex (Ohzawa et al., 1985; Carandini, 2000; Dhruv et al., 2011). Furthermore, hyperpolarization of the membrane potential has been associated with contrast adaptation (Carandini and Ferster, 1997; Sanches-Vives et al., 2000a,b). However, questions about the specific mechanisms involved, their relative contributions, and the stage each one is implemented remain unanswered. It seems that some of the investigative tools currently most readily applied in the mouse could provide insights into these cellular and circuit mechanisms. The same biochemical and genetic flexibility that has allowed the use of optogenetic modulation to attribute particular functions to genetically defined inhibitory neurons within mouse V1 (e.g., Adesnik et al., 2012; Atallah et al., 2012), or allowed genetically encoded calcium-indicator proteins to explore the response properties of hundreds of visually responsive neurons simultaneously (e.g., Andermann et al., 2011), could also be used to explore the mechanisms underlying contrast adaptation. Moreover, if specific mechanisms are isolated they could be knocked-out or modulated in real-time to probe the perceptual relevance of contrast adaptation using psychophysical tasks developed for the mouse (e.g., Busse et al., 2011). The possibility of causally linking neural processing to contrast perception is especially intriguing considering psychophysical studies of the performance enhancement conferred by contrast adaptation have been somewhat equivocal (Barlow et al., 1976; Määttänen and Koenderink, 1991; Abbonizio et al., 2002; Kohn, 2007). We are hopeful that insights gleaned from the mouse model will be relevant to higher mammals because the properties of cortical contrast adaptation that have already been explored appear quite similar between species.

## Author contributions

Nathan A. Crowder and Emily E. LeDue designed the study; Emily E. LeDue, Jillian L. King, Kurt R. Stover, and Nathan A. Crowder collected data; Emily E. LeDue, Jillian L. King, and Nathan A. Crowder analyzed the data; Nathan A. Crowder and Emily E. LeDue wrote the manuscript.

### Conflict of interest statement

The authors declare that the research was conducted in the absence of any commercial or financial relationships that could be construed as a potential conflict of interest.

## References

[B1] Abbonizio G. Langley K. Clifford C. W. (2002). Contrast adaptation may enhance contrast discrimination. Spat. Vis. 16, 45–58 10.1163/15685680260433904 12636224

[B2] Adesnik H. Bruns W. Taniguchi H. Huang Z. J. Scanziani M. (2012). A neural circuit for spatial summation in visual cortex. Nature 490, 226–231 10.1038/nature11526 23060193 PMC3621107

[B3] Albrecht D. G. Farrar S. B. Hamilton D. B. (1984). Spatial contrast adaptation characteristics of neurones recorded in the cat's visual cortex. J. Physiol. 347, 713–739 6707974 10.1113/jphysiol.1984.sp015092 PMC1199473

[B4] Albrecht D. G. Hamilton D. B. (1982). Striate cortex of monkey and cat: contrast response function. J. Neurophysiol. 48, 217–237 7119846 10.1152/jn.1982.48.1.217

[B5] Andermann M. L. Kerlin A. M. Roumis D. K. Glickfield L. L. Reid R. C. (2011). Functional specialization of mouse higher visual cortical areas. Neuron 72, 1025–1039 10.1016/j.neuron.2011.11.013 22196337 PMC3876958

[B6] Atallah B. V. Bruns W. Carandini M. Scanziani M. (2012). Parvalbumin-expressing interneurons linearly transform cortical responses to visual stimuli. Neuron 73, 159–170 10.1016/j.neuron.2011.12.013 22243754 PMC3743079

[B7] Barlow H. B. Macleod D. I. van Meeteren A. (1976). Adaptation to gratings: no compensatory advantages found. Vision Res. 16, 1043–1045 10.1016/0042-6989(76)90241-8 969214

[B8] Blakemore C. Campbell F. W. (1969). Adaptation to spatial stimuli. J. Physiol. 200, 11–13 5761934 PMC1350050

[B9] Blakemore C. Muncey J. P. Ridley R. M. (1973). Stimulus specificity in the human visual system. Vision Res. 13, 1915–1931 10.1016/0042-6989(73)90063-1 4746989

[B10] Blakemore C. Nachmias J. (1971). The orientation specificity of two visual after-effects. J. Physiol. 213, 157–174 5575335 10.1113/jphysiol.1971.sp009374 PMC1331729

[B11] Bonds A. B. (1991). Temporal dynamics of contrast gain in single cells of the cat striate cortex. Vis. Neurosci. 6, 239–255 10.1017/S0952523800006258 2054326

[B12] Brainard D. H. (1997). The psychophysics toolbox. Spat. Vis. 10, 433–436 10.1163/156856897X00357 9176952

[B13] Busse L. Ayaz A. Dhruv N. T. Katzner S. Saleem A. B. Schölvinck M. L. (2011). The detection of visual contrast in the behaving mouse. J. Neurosci. 31, 11351–11361 10.1523/JNEUROSCI.6689-10.2011 21813694 PMC6623377

[B14] Carandini M. (2000). Visual cortex: fatigue and adaptation. Curr. Biol. 10, 605–607 10.1016/S0960-9822(00)00637-0 10985379

[B15] Carandini M. Demb J. B. Mante V. Tolhurst D. J. Dan Y. Olshausen B. A. (2005). Do we know what the early visual system does? J. Neurosci. 25, 10577–10597 10.1523/JNEUROSCI.3726-05.2005 16291931 PMC6725861

[B16] Carandini M. Ferster D. (1997). A tonic hyperpolarization underlying contrast adaptation in cat visual cortex. Science 276, 949–952 10.1126/science.276.5314.949 9139658

[B17] Crowder N. A. Hietanen M. A. Price N. S. C. Clifford C. W. G. Ibbotson M. R. (2008). Dynamic contrast change produces rapid gain control in visual cortex. J. Physiol. 586, 4107–4119 10.1113/jphysiol.2008.156273 18599535 PMC2652193

[B18] Crowder N. A. Price N. S. C. Hietanen M. A. Dreher B. Clifford C. W. G. Ibbotson M. R. (2006). Relationship between contrast adaptation and orientation tuning in V1 and V2 of cat visual cortex. J. Neurophysiol. 95, 271–283 10.1152/jn.00871.2005 16192327

[B19] Crowder N. A. van Kleef J. Dreher B. Ibbotson M. R. (2007). Complex cells increase their phase sensitivity at low contrasts and following adaptation. J. Neurophysiol. 98, 1155–1166 10.1152/jn.00433.2007 17537901

[B20] DeAngelis G. C. Uka T. (2003). Coding of horizontal disparity and velocity by MT neurons in the alert macaque. J. Neurophysiol. 89, 1094–1111 10.1152/jn.00717.2002 12574483

[B21] Demb J. B. (2008). Functional circuitry of visual adaptation in the retina. J. Physiol. 586, 4377–4384 10.1113/jphysiol.2008.156638 18617564 PMC2614018

[B22] Derrington A. M. Lennie P. (1984). Spatial and temporal contrast sensitivities of neurones in lateral geniculate nucleus of macaque. J. Physiol. 357, 219–240 6512690 10.1113/jphysiol.1984.sp015498 PMC1193256

[B23] Dhruv N. T. Tailby C. Sokol S. H. Lennie P. (2011). Multiple adaptable mechanisms early in the primate visual pathway. J. Neurosci. 31, 15016–15025 10.1523/JNEUROSCI.0890-11.2011 22016535 PMC3271438

[B24] Duong T. Freeman R. D. (2007). Spatial frequency-specific contrast adaptation originates in the primary visual cortex. J. Neurophysiol. 98, 187–195 10.1152/jn.01364.2006 17428911

[B25] Foley J. M. Chen C. C. (1997). Analysis of the effect of pattern adaptation on pattern pedestal effects: a two-process model. Vision Res. 37, 2779–2788 10.1016/S0042-6989(97)00081-3 9373676

[B26] Gao E. DeAngelis G. C. Burkhalter A. (2010). Parallel input channels to mouse primary visual cortex. J. Neurosci. 30, 5912–5926 10.1523/JNEUROSCI.6456-09.2010 20427651 PMC3129003

[B27] Greenlee M. W. Heitger F. (1988). The functional role of contrast adaptation. Vision Res. 28, 791–797 10.1016/0042-6989(88)90026-0 3227656

[B28] Grubb M. S. Thompson I. D. (2003). Quantitative characterization of visual response properties in the mouse dorsal lateral geniculate nucleus. J. Neurophysiol. 90, 3594–3607 10.1152/jn.00699.2003 12944530

[B29] Hawken M. J. Shapley R. M. Grosof D. H. (1996). Temporal-frequency selectivity in monkey visual cortex. Vis. Neurosci. 13, 477–492 10.1017/S0952523800008154 8782375

[B30] Henry C. A. Hawken M. J. (2013). Stability of simple/complex classification with contrast and extraclassical receptive field modulation in macaque V1. J. Neurophysiol. 109, 1793–1803 10.1152/jn.00997.2012 23303859 PMC3628017

[B31] Hietanen M. A. Cloherty S. L. van Kleef J. P. Wang C. Dreher B. Ibbotson M. R. (2013). Phase sensitivity of complex cells in the primary visual cortex. Neuroscience 237, 19–28 10.1016/j.neuroscience.2013.01.030 23357120

[B32] Ibbotson M. R. (2005). “Physiological mechanisms of adaptation in the visual system,” in Fitting the Mind to the World: Adaptation and After-Effects in High Level Vision, ed Clifford C. W. G. Rhodes G. (Oxford: Oxford University Press), 15–45 10.1093/acprof:oso/9780198529699.003.0002

[B33] Kaneko M. Xie Y. An J. J. Stryker M. P. Xu B. (2012). Dendritic BDNF synthesis is required for late-phase spine maturation and recovery of cortical responses following sensory deprivation. J. Neurosci. 32, 4790–4802 10.1523/JNEUROSCI.4462-11.2012 22492034 PMC3356781

[B34] Kohn A. (2007). Visual adaptation: physiology, mechanisms, and functional benefits. J. Neurophysiol. 97, 3155–3164 10.1152/jn.00086.2007 17344377

[B35] LeDue E. E. Zou M. Y. Crowder N. A. (2012). Spatiotemporal tuning in mouse primary visual cortex. Neurosci. Lett. 528, 165–169 10.1016/j.neulet.2012.09.006 22995183

[B36] Määttänen L. M. Koenderink J. J. (1991). Contrast adaptation and contrast gain control. Exp. Brain Res. 87, 205–212 10.1007/BF00228521 1756826

[B37] Marshel J. H. Garrett M. E. Nauhaus I. Callaway E. M. (2011). Functional specialization of seven mouse visual cortical areas. Neuron 72, 1040–1054 10.1016/j.neuron.2011.12.004 22196338 PMC3248795

[B38] Mechler F. Ringach D. L. (2002). On the classification of simple and complex cells. Vision Res. 42, 1017–1033 10.1016/S0042-6989(02)00025-1 11934453

[B39] Moldestad O. Karlsen P. Molden S. Storm J. F. (2009). Tracheotomy improves experiment success rate in mice during urethane anesthesia and stereotaxic surgery. J. Neurosci. Methods 176, 57–62 10.1016/j.jneumeth.2008.08.015 18778735

[B40] Movshon J. A. Lennie P. (1979). Pattern-selective adaptation in visual cortical neurones. Nature 278, 850–852 10.1038/278850a0 440411

[B41] Movshon J. A. Thompson I. D. Tolhurst D. J. (1978a). Spatial summation in the receptive fields of simple cells in the cat's striate cortex. J. Physiol. 283, 57–77 722589 10.1113/jphysiol.1978.sp012488 PMC1282765

[B42] Movshon J. A. Thompson I. D. Tolhurst D. J. (1978b). Receptive field organization of complex cells in the cat's striate cortex. J. Physiol. 283, 79–99 722592 10.1113/jphysiol.1978.sp012489 PMC1282766

[B43] Müller J. R. Metha A. B. Krauskopf J. Lennie P. (1999). Rapid adaptation in visual cortex to the structure of images. Science 285, 1405–1408 10.1126/science.285.5432.1405 10464100

[B44] Niell C. M. Stryker M. P. (2008). Highly selective receptive fields in mouse visual cortex. J. Neurosci. 28, 7520–7536 10.1523/JNEUROSCI.0623-08.2008 18650330 PMC3040721

[B45] Ohzawa I. Sclar G. Freeman R. D. (1982). Contrast gain control in the cat visual cortex. Nature 298, 266–268 10.1038/298266a0 7088176

[B46] Ohzawa I. Sclar G. Freeman R. D. (1985). Contrast gain control in the cat's visual system. J. Neurophysiol. 54, 651–667 4045542 10.1152/jn.1985.54.3.651

[B47] Paxinos G. Franklin K. B. J. (2001). The Mouse Brain in Stereotaxic Coordinates. New York, NY: Academic

[B48] Pelli D. G. (1997). The video toolbox software for visual psychophysics: transforming numbers into movies. Spat. Vis. 10, 437–442 10.1163/156856897X00366 9176953

[B49] Price N. S. Ono S. Mustari M. J. Ibbotson M. R. (2005). Comparing acceleration and speed tuning in macaque MT: physiology and modeling. J. Neurophysiol. 94, 3451–3464 10.1152/jn.00564.2005 16079192

[B50] Sanches-Vives M. V. Nowak L. G. McCormick D. A. (2000a). Membrane mechanisms underlying contrast adaptation in cat area 17 *in vivo*. J. Neurosci. 20, 4267–4285 10818163 10.1523/JNEUROSCI.20-11-04267.2000 PMC6772627

[B51] Sanches-Vives M. V. Nowak L. G. McCormick D. A. (2000b). Cellular mechanisms of long-lasting adaptation in visual cortical neurons *in vitro*. J. Neurosci. 20, 4286–4299 10818164 10.1523/JNEUROSCI.20-11-04286.2000 PMC6772630

[B52] Saul A. B. Cynader M. S. (1989a). Adaptation in single units in visual cortex: the tuning of aftereffects in the spatial domain. Vis. Neurosci. 2, 593–607 10.1017/S0952523800003527 2487086

[B53] Saul A. B. Cynader M. S. (1989b). Adaptation in single units in visual cortex: the tuning of aftereffects in the temporal domain. Vis. Neurosci. 2, 509–620 10.1017/S0952523800003539 2487087

[B54] Sclar G. Lennie P. DePriest D. D. (1989). Contrast adaptation in striate cortex of macaque. Vision Res. 29, 747–755 10.1016/0042-6989(89)90087-4 2623819

[B55] Skottun B. C. De Valois R. L. Grosof D. H. Movshon J. A. Albrecht D. G. Bonds A. B. (1991). Classifying simple and complex cells on the basis of response modulation. Vision Res. 31, 1079–1086 10.1016/0042-6989(91)90033-2 1909826

[B56] Snowden R. J. Hammett S. T. (1996). Spatial frequency adaptation: threshold elevation and perceived contrast. Vision Res. 36, 1797–1809 10.1016/0042-6989(95)00263-4 8759448

[B57] Solomon S. G. Pierce J. W. Dhruv N. T. Lennie P. (2004). Profound contrast adaptation early in the visual pathway. Neuron 42, 155–162 10.1016/S0896-6273(04)00178-3 15066272

[B58] Stroud A. C. LeDue E. E. Crowder N. A. (2012). Orientation specificity of contrast adaptation in mouse primary visual cortex. J. Neurophysiol. 108, 1381–1391 10.1152/jn.01148.2011 22696541

[B59] Van den Bergh G. Zhang B. Arckens L. Chino Y. M. (2010). Receptive-field properties of V1 and V2 neurons in mice and macaque monkeys. J. Comp. Neurol. 518, 2051–2070 10.1002/cne.22321 20394058 PMC2881339

[B60] Vautin R. G. Berkley M. A. (1977). Responses of single cells in cat visual cortex to prolonged stimulus movement: neural correlates of visual aftereffects. J. Neurophysiol. 40, 1051–1065 903797 10.1152/jn.1977.40.5.1051

